# If laparoscopic technique can be used for treatment of acutely incarcerated/strangulated inguinal hernia?

**DOI:** 10.1186/s13017-021-00348-1

**Published:** 2021-02-06

**Authors:** Jing Liu, Yingmo Shen, Yusheng Nie, Xuefei Zhao, Fan Wang, Jie Chen

**Affiliations:** Department of Hernia and Abdominal Wall Surgery, Beijing Chaoyang Hospital, Capital Medical University, Number 5 Jingyuan Road, Shijingshan District, Beijing, 100043 China

**Keywords:** Incarcerated/strangulated inguinal hernia, Transabdominal preperitoneal repair, Laparoscopy

## Abstract

**Purpose:**

Laparoscopic treatment for acutely incarcerated**/**strangulated inguinal hernias is uncommon and controversial. In the present study, we assessed the safety and feasibility of transabdominal preperitoneal (TAPP) repair for the treatment of acutely incarcerated**/**strangulated inguinal hernias.

**Methods:**

Patients with acutely incarcerated**/**strangulated inguinal hernias who underwent TAPP repair at the Department of Hernia and Abdominal Wall Surgery (Beijing Chaoyang Hospital) from January 2017 to December 2019 were retrospectively reviewed. Patients’ characteristics, operation details, and postoperative complications were retrospectively analyzed.

**Results:**

In total, 94 patients with acutely incarcerated**/**strangulated inguinal hernias underwent TAPP repair. The patients comprised 85 men and 9 women (mean age, 54.3 ± 13.6 years; mean operating time, 61.6 ± 17.7 min; mean hospital stay, 3.9 ± 2.2 days). No patients were converted to open surgery. Hernia reduction was successfully performed in all patients. The morbidity of complications was 20.2% (19/94). Two bowel resections were performed endoscopically. Nine (9.6%) patients avoided unnecessary bowel resections during laparoscopic procedures. All patients recovered well without severe complications. No recurrence or infection was recorded during a mean follow-up period of 26.8 ± 9.8 months.

**Conclusions:**

TAPP appears to be safe and feasible for treatment of patients with acutely incarcerated**/**strangulated inguinal hernias. However, it requires performed by experienced surgeons in laparoscopic techniques.

## Introduction

Acutely incarcerated inguinal hernia is a common acute abdominal disease. The probability of strangulation is 0.29% in all inguinal hernias [1]. The mortality rate may reach about 5% among patients of advanced age who present on an emergency basis [2, 3]. Patients diagnosed with acutely incarcerated/strangulated inguinal hernia require rapid surgical intervention. Transabdominal preperitoneal (TAPP) hernia repair has been proven effective [4–6] in selected inguinal hernia repairs. However, this approach is uncommon and controversial in the treatment of acutely incarcerated/strangulated inguinal hernias. In this study, we evaluated the safety and feasibility of TAPP repair in the treatment of acutely incarcerated/strangulated inguinal hernias.

## Methods

We retrospectively reviewed 94 patients with acutely incarcerated/strangulated inguinal hernias who were treated by emergency TAPP from January 2017 to December 2019 at our hospital (Department of Hernia and Abdominal Wall Surgery, Beijing Chaoyang Hospital, Capital Medical University). The patients’ characteristics, operative details, duration of hospital stay, incidence of complications, mortality, and recurrence were reviewed.

The criteria for evaluating the feasibility of the laparoscopic technique were the mean operative time, ability to reduce the hernia, rate of conversion to open surgery, and diagnostic rate of contralateral inguinal hernia. The criteria for evaluating the safety of the laparoscopic technique were the mean hospital stay, mortality, morbidity of complications, and hernia recurrence rate.

The inclusion criteria were adult patients with incarcerated/strangulated inguinal hernias, no previous severe abdominal surgery, no abdominal wall infection, no severe cardiopulmonary function diseases, and a body condition fit for general anesthesia. The exclusion criteria were patients with contraindications for general anesthesia, signs of peritonitis, a definitive diagnosis of bowel perforation before surgery, and severe bowel distension preventing the use of a laparoscopic technique.

### Surgical technique

All operations were performed by a single surgeon, and TAPP repair was performed in all cases. After inducing general anesthesia, pneumoperitoneum was established with a Veress needle up to 12 to 14 mmHg. A 10-mm trocar was inserted into the abdominal cavity through the incision just below the umbilicus. A 30° video camera was placed into the abdominal cavity through the 10-mm trocar. Under laparoscopic visualization, two 5-mm trocars were placed at each midclavicular line (1 or 2 cm under the umbilicus). First, we detected the inguinal region and evaluated the hernia contents. The next step was hernia reduction, which involved some technical difficulties. Hernia reduction might be assisted by general anesthesia. If direct traction failed, manual pressure to inguinal region from outside could be applied by an assistant. If the above methods failed, enlargement of the hernia ring with an electronic hook became necessary. The hernia ring was incised ventrolaterally for indirect hernias and ventromedially for direct hernias. After successful reduction, the vitality of the hernia content could be clearly detected. Standard TAPP repair was then performed [7]. Severe incarcerated bowel was detected again in the last step, and bowel resection was performed endoscopically if necessary. We converted to open surgery in patients with bowel perforation. Any accompanying contralateral inguinal hernias were simultaneously repaired by the TAPP approach.

Presentation of incarcerated or strangulated inguinal hernia includes a non-reducible mass of the inguinal region or scrotum in both sanding and supine positions. Localized tenderness or pain on examination frequently reported in patients. Some patients have gastrointestinal signs and symptoms. Any continuous or severe discomfort, erythema of skin, nausea, or vomiting associated with the bulge are signs that the hernia may be strangulated. Patients were assessed by lab tests, ultrasound (US), and computed tomography (CT) examinations in the preoperative time. US and CT are valuable examinations to definite the location, size, and the contents within the hernia sac. The type of mesh used was monofilament polypropylene mesh, which is lightweight with large pores. All meshes were fixed with medical glue (n-butyl-cyanoacrylate; Compont Medical Devices, Beijing, China). The peritoneum was closed with absorbable suture (Vicryl 3/0, Ethicon).

All patients were given a perioperative prophylactic antibiotic (cefuroxime sodium). If the skin test was positive, levofloxacin was used. The patients’ clinical data were reviewed, and all patients were followed up by telephone calls.

## Results

During the study period, we received a total 269 patients with acutely incarcerated/strangulated inguinal hernias in our department. Ninety-four patients who fulfilled the criteria were recruited in our study, 14 patients with TAPP were lost to follow-up and the other 161 patients who underwent open surgeries were excluded from our study. The patients’ characteristics and operative details are shown in Table 1. The patients comprised 85 men and 9 women with a mean age of 54.3 ± 13.6 years (range, 21–75 years). The mean BMI was 25.1 ± 2.4 kg/m^2^(range, 21–35). The mean duration of symptoms was 9.7 ± 7.5 h (range, 3–48 h). An indirect inguinal hernia was present in 73 (77.7%) patients, and a direct inguinal hernia was present in 21 (22.3%). The mean operative time was 61.6 ± 17.7 min (range, 35–120 min), and the mean hospitalization was 3.9 ± 2.2 days (range, 1–12 days).

**Table 1:** Patients’ characteristics and operative details

Items	Results
Age, years	
Mean ± SD	54.3 ± 13.6
Range	21–75
Sex	
Male	85(90.4%)
Female	9(9.6%)
Hernia type	
Indirect	73(77.7%)
Direct	21(22.3%)
BMI, kg/m^2^	
Mean ± SD	25.1 ± 2.4
Range	21–35
Duration of symptoms, h	
Mean ± SD	9.7 ± 7.5
Range	3–48
Operating times, min	
Mean ± SD	61.6 ± 17.7
Range	35–120
Hospitalization, days	
Mean ± SD	3.9 ± 2.2
Range	1–12
Visceral resection	5 (5.3%)
Omentum	3 (3.2%)
Bowel	2 (2.1%)
Contralateral hernias	15(15.9%)
Unnecessary bowel resection	9(9.6%)
Conversion to open	0(0)

The total resection rate was 5.3% (5/94). Two (2.1%) bowel resections and 3 (3.2%) omental resections were performed due to strangulation. All resections were performed endoscopically. After laparoscopic exploration, 15 (15.9%) contralateral hernias were diagnosed and simultaneously repaired by the same TAPP procedure. Nine (9.6%) patients were highly suspected to have had necrotic bowel avoided unnecessary bowel resections because the vitality of the incarcerated bowel recovered to normal after the TAPP procedure. No patients were converted to open surgery.

The hernia content was small intestine in 51 (54.2%) patients, omental tissue in 36 (38.3%), colon in 6 (6.4%), and urinary bladder in 1 (1.1%). All hernia contents were successfully reduced. Hernia reduction occurred spontaneously after general anesthesia in 20 (21.3%) patients, direct traction with manual compression occurred in 32(34%), and incision of the hernia ring occurred in 42 (44.7%) (Table 2).

**Table 2:** Approaches of hernia reduction

Type	*N*(%)
Reduction during anesthesia	20(21.3)
Direct traction with manual compression	32(34)
Incision of hernia ring	42(44.7)

Complications are shown in Table 3. No mortality occurred in this study. The morbidity rate associated with complications was 20.2% (19/94). One (1.1%) patient sustained an abdominal wall vascular injury during trocar insertion and finally recovered uneventfully by ligation. No other injuries or intraoperative complications occurred. According to Clavien–Dindo classification, Grade I complications were recorded in all 18 (19.1%) patients: 3 (3.2%) developed urine retention, and 15 (15.9%) developed a scrotal seroma. Grade II–IV complications were not recorded in our study. All the seromas were cured within 2 months by conservative treatment. No wound or mesh infections occurred.

**Table 3:** Major complications

Complications	*N* (%)
Total	19 (20.2)
Vessel injury	1 (1.1)
Seroma/hematoma	15(15.9)
Urine retention	3 (3.2)
Visceral injury	0(0)
Wound infection	0(0)
Mesh infection	0(0)
Lesion of spermatic structure	0(0)
Pulmonary	0(0)
Cardiovascular	0(0)
Thrombosis	0(0)
Recurrence	0(0)
Mortality	0(0)

The follow-up rate was 90%. During a mean follow-up time of 26.8 ± 9.8 months (range, 6–42 months), no further infections occurred. No recurrence or mortality occurred during the follow-up period.

## Discussion

The safety and efficacy of laparoscopy have been proven in selective operations [4, 8, 9]. However, the use of this approach in emergency cases is still controversial. The first treatment of an incarcerated inguinal hernia with a laparoscopic technique was reported in 1993 [10]. TAPP repair was gradually accepted thereafter. In 2013, the European Association for Endoscopic Surgery concluded that laparoscopy can be applied for treatment of incarcerated inguinal hernias [11]. However, relevant studies are scarce, and the applications of TAPP repair remain largely debated.

Some authors have advocated the performance of hernia reduction under laparoscopic vision to increase the accuracy and safety of the procedure [12, 13]. Moreover, the laparoscopic approach allows for a more thorough internal abdomen exploration to evaluate organ vitality and provides sufficient time to make decisions regarding bowel resection. Another advantage of laparoscopy is that it facilitates diagnosis and treatment of contralateral hernias. The main challenges associated with laparoscopic emergency hernia repair are technical difficulties in dissection of the hernia sac, difficulties in hernia reduction through a narrowed inguinal ring, and an increased risk of iatrogenic injuries [14, 15].

No specific parameters are available to estimate the technical difficulties of TAPP repair [13]. Special techniques should be focused on hernia reduction. In our experience, hernia reduction has some technical key points. General anesthesia usually benefits hernia reduction. In some cases, the hernia contents reduce spontaneously after general anesthesia, while in others the hernia contents are reduced by direct traction or with manual compression from outside. Blind traction is forbidden. If this procedure fails, enlargement of the hernia ring facilitates hernia reduction. Our data revealed that hernia ring incision is a good means of hernia reduction, resulting in an easy reduction of hernia contents without additional injuries. We usually incise the hernia ring with an electronic hook in the preperitoneal plane. The peritoneum can provide protection to the incarcerated small bowel from iatrogenic injuries caused by the electronic hook. In our experience, it is recommended that the hernia ring is cut ventrolaterally for indirect hernias and ventromedially for direct hernias to avoid injury of the inferior epigastric artery. Other authors have stated similar concerns [16]. Naturally, all procedures must be performed with great care. The use of non-traumatic forceps for hernia reduction is a good choice.

After hernia reduction, the most important step is to assess the vitality of the hernia contents. Our result revealed that the most frequently encountered content in incarcerated hernia was small intestine. If a definite diagnosis cannot be immediately made, TAPP repair can be performed first. Surgery can provide sufficient observation time to make a decision about bowel resection. In some cases, the color of suspected ischemic bowel may recover to normal after hernia repair, thus avoiding unnecessary bowel resection. In our study, bowel resection was only performed in two patients. Unnecessary bowel resections were avoided in nine patients because the color of the incarcerated bowel recovered to normal after the TAPP procedure. Similar results have been reported in other previously published studies [16–18].

Laparoscopy provides a good view of the internal abdominal cavity, making it a useful diagnostic technique, especially for those with hernia contents that reduced spontaneously preoperatively. Once the potentially necrotic bowel has spontaneously reduced into the abdominal cavity, the patient may develop bowel perforation, abdominal infection, or even life-threatening sepsis. We can detect the reduced bowel via laparoscopy to avoid these problems. Furthermore, a contralateral inguinal hernia can be diagnosed and repaired simultaneously via laparoscopy.

The conversion rate is one of the criteria used to evaluate the feasibility of the laparoscopic technique. No conversion occurred in our study, suggesting that TAPP repair is feasible for treatment of acutely incarcerated/strangulated inguinal hernias. Other previously published papers also support our result [13, 16, 17, 19]. Bowel distension was recognized as the reason for conversion [12]. No conversion occurred in our study. The reasons were as follows: first, our patients were strictly selected. Patients with severe bowel distension or patients who had signs of bowel gangrene with abdominal wall infection or peritonitis were excluded; second, all procedures must be performed with great care. Blind traction was forbidden to prevent iatrogenic bleeding or bowel injury. Third, the hernia ring must be cut in patients with severe incarceration. This additional procedure can quickly release the incarceration, resulting in an easy hernia reduction. Furthermore, the surgeon’s ability will determine whether the laparoscopic technique can be successfully performed in emergency cases because this technique involves a long learning curve [12]. TAPP repair are recommended to be performed by senior surgeons who have gained sufficient experience in laparoscopic techniques.

Mesh repair for patients with acute hernias can decrease the risk of recurrence [20–23]. However, the use of mesh in the treatment of acute hernias remains controversial. Mesh implantation has been considered a potential risk factor for the development of infection [24, 25]. However, there is no sufficient evidence to support this assumption. The safety of mesh repair for acute hernias had been proven in published studies with favorable results [26–30]. Our results also did not support the assumption that mesh implantation is a risk factor for infection. In our study, all patients underwent repair with mesh, and no infection occurred.

Mesh infection is a devastating complication that is difficult to eradicate without mesh removal. The risk of infection is mainly associated with the type of filament used and the pore size [31]. Multifilament meshes are more prone to infection than monofilament meshes [32, 33]. Lightweight meshes have large pores. They are superior because of their greater elasticity, higher flexibility, and reduced discomfort [31, 34]. Larger pores are also associated with decreased inflammation [35]; moreover, they shrink less and have a reduced risk of infection [31]. We recommend to choose the light mesh with large pore for implantation. All of our patients underwent repair with lightweight monofilament polypropylene mesh with large pores. No mesh infection occurred.

The use of prophylactic antibiotics is advisable. Consistent with the views of other authors [16, 36], peritoneum irrigation can be performed after hernia reduction. Mesh implantation is contraindicated when the bowel gangrene or perforation occurs. During our follow-up period of 26.8 ± 9.8 months, no recurrence or additional infections were recorded.

The most frequently observed postoperative complication in this study was a scrotal seroma. All seromas developed below the external ring and under the superficial soft tissue of the groin or the scrotum, where the hernia sac was located [37]. The reported incidence of seroma formation varies from 0.5 to 12.2% [38]. Studies have shown that the incidence of seroma formation is significantly higher after laparoscopic hernia repair [9, 39, 40]. The incidence in the present study was slightly higher (15.9%). However, this was not a severe problem, and it resolved spontaneously within 8 weeks. In our opinion, only symptomatic seromas should be treated.

One of our patients underwent abdominal wall vascular injury during the process of trocar insertion. This accident occurred in the early stage of laparoscopy application in our department and was remedied by ligation with no further problems. No further injury or other intraoperative complications occurred.

The laparoscopic approach may not be applicable to all emergency cases. A consensus has not yet been reached. Based on our evaluation and experience, we consider that TAPP repair can be safely performed in strictly selected patients with acutely incarcerated/strangulated inguinal hernias. We look forward to obtaining further long-term results in the future.

## Conclusions

TAPP repair is safe and feasible in the treatment of selected patients with acutely incarcerated/strangulated inguinal hernias. However, it requires to be performed by surgeons experienced in laparoscopic techniques.

## Data Availability

The data sets supporting the results of this article are included within the article and its additional files.

## Abbreviations

TAPP: Transabdominal preperitoneal
US: Ultrasound
CT: Computed tomography
